# Risk prediction of CMV reactivation after allogeneic stem cell transplantation using five non-HLA immunogenetic polymorphisms

**DOI:** 10.1007/s00277-022-04841-8

**Published:** 2022-05-07

**Authors:** Miren Vallejo, Paula Muñiz, Mi Kwon, Laura Solán, Rebeca Bailén, Diego Carbonell, María Chicano, Julia Suárez-González, Pilar Catalán, José María Bellón, Juan Carlos Triviño, Nieves Dorado, David Gallardo, José Luis Díez-Martín, Natalia Ramírez, Carolina Martínez-Laperche, Ismael Buño

**Affiliations:** 1 Oncohematology Research Group, Navarrabiomed, Complejo Hospitalario de Navarra, Universidad Pública de Navarra (UPNA), Navarra Institute for Health Research (IdiSNA), Pamplona, Spain; 2 Department of Hematology, Gregorio Marañón General University Hospital, Madrid, Spain; 3 Gregorio Marañón Health Research Institute (IiSGM), C/Doctor Esquerdo 46, 28007 Madrid, Spain; 4 Genomics Unit, Gregorio Marañón General University Hospital, IiSGM, Madrid, Spain; 5 Department of Microbiology, Gregorio Marañón General University Hospital, Madrid, Spain; 6 Sistemas Genómicos, Valencia, Spain; 7 Clinical Hematology Department, Institut Català d’Oncologia (ICO Girona), Girona, Spain; 8 Department of Medicine, School of Medicine, Complutense University of Madrid, Madrid, Spain; 9 Department of Cell Biology, School of Medicine, Complutense University of Madrid, Madrid, Spain

**Keywords:** Allogeneic hematopoietic stem cell transplant, Cytomegalovirus reactivation, Immune gene, Polymorphisms

## Abstract

Despite advances in the understanding of the pathophysiology of cytomegalovirus (CMV) infection, it remains as one of the most common infectious complications after allogeneic hematopoietic stem cell transplantation (allo-HSCT). The aim of this study was to determine the genotype of cytokines and chemokines in donor and recipient and their association with CMV reactivation. Eighty-five patients receiving an allo-HSCT from an HLA-identical sibling donor were included in the study. Fifty genes were selected for their potential role in the pathogenesis of CMV infection. CMV DNAemia was evaluated until day 180 after allo-HSCT. CMV reactivation was observed in 51/85 (60%) patients. Of the 213 genetic variants selected, 11 polymorphisms in 7 different genes (CXCL12, IL12A, KIR3DL1, TGFB2, TNF, IL1RN, and CD48) were associated with development or protection from CMV reactivation. A predictive model using five of such polymorphisms (*CXCL12* rs2839695, *IL12A* rs7615589, *KIR3DL1* rs4554639, *TGFB2* rs5781034 for the recipient and *CD48* rs2295615 for the donor) together with the development of acute GVHD grade III/IV improved risk stratification of CMV reactivation. In conclusion, the data presented suggest that the screening of five polymorphisms in recipient and donor pre-transplantation could help to predict the individual risk of CMV infection development after HLA-identical allo-HSCT.

**Supplementary Information:**

The online version contains supplementary material available at 10.1007/s00277-022-04841-8.

## Introduction

Cytomegalovirus (CMV) infection is one of the most common viral infections after allogeneic hematopoietic stem cell transplantation (allo-HSCT). In patients without CMV prophylaxis and depending on the transplant setting, incidences of CMV reactivation after allo-HSCT among CMV-seropositive patients are as high as 80%. In addition to the direct effects of CMV reactivation, tissue-invasive CMV disease may be associated with increased risk of graft-versus-host-disease (GVHD) and infections [1]. T-cell mediated cellular immunity is the most important factor in controlling CMV replication. CMV induces a strong CD8 + cytotoxic T-lymphocyte (CTL) response; therefore, immunosuppression significantly contributes to the loss of CMV specific adaptive immune control [2, 3]. However, the observation that only a fraction of patients with similar degrees of immunosuppression develops CMV infection suggests that other factors not yet defined contribute to susceptibility to reactivations.

Cytokines and chemokines are the first line of defense against viral infections [4]. High post-transplant proinflammatory cytokine levels have been associated with the risk for developing CMV infection [5]. Recent studies demonstrated that cytokine gene polymorphisms result in inter-individual differences in cytokine production [6]. To date, several groups have demonstrated that genetic differences, particularly single-nucleotide polymorphisms (SNPs), in non-HLA genes between recipients and donors influence transplant outcome and can be used as biomarkers to anticipate post-transplantation complications such as GVHD [7]. With the development of next-generation sequencing methods, an extensive genetic characterization of donor and recipient is possible in order to determine the contributions of a large number of different polymorphisms in immune-related genes to the allo-HSCT outcomes.

Numerous reports have also demonstrated that polymorphisms in different genes influence the outcome and course of infections, particularly CMV infection [8, 9]. SNPs in genes coding for cytokines or chemokines have been reported to be associated with increased risks for CMV infections [10–14]. Such polymorphisms may influence the rate and regulatory dynamics of gene transcription, the stability of the mRNA, and the production and biological activity of the resulting protein. Corrales et al. reported that patients carrying the *CCR5* A/A genotype displayed episodes of active CMV infection with higher CMV viral load [12]. Other authors have found that heterozygosity for the toll-like receptor (*TLR*) 2 and *TLR4* SNPs was associated with lower risk of CMV infection and lower level of viremia, respectively and, therefore, these polymorphisms appear to be protective factors in CMV replication [13]. In addition, allo-HSCT recipients that experienced active CMV infection usually present higher frequencies of the genotype T/T of the *CD28* gene. Recently, the genotype of the donor-activating killer immunoglobulin-like receptor (*KIR)*, which regulates NK cell function, has been demonstrated to influence the development of CMV infection after allo-HSCT [15]. Two studies showed that allo-HSCT recipients of donor haplotypes containing the activating *KIR2DS2* and *KIR2DS4* genes had a reduced risk of CMV infection compared to those who received grafts with other haplotypes [16, 17].

Therefore, there are increasing evidences indicating that polymorphisms in genes coding for cytokines or chemokines and their receptors may modulate the susceptibility to, as well as the dynamics and outcomes, of CMV infections. The present study aims to determine whether the genotype of the donor and recipient for 50 immune-system related genes influences CMV reactivation in patients receiving an allo-HSCT from an HLA-identical sibling donor.

## Methods

### Study design

Ninety consecutive patients who received allo-HSCT from an HLA-identical sibling donor from 2000 to 2015 at Gregorio Marañón General University Hospital (HGUGM) and with available sample for analysis were included in the study. Simultaneously, their sibling donors (*n* = 90) were also analyzed. The study period comprised the first 180 days following allo-HSCT. However, five patients (5.4%) who died before day 180 without CMV reactivation were excluded from the analyses. The ethics committee of HGUGM approved the study. All recipients and donors provided written informed consent according to the Declaration of Helsinki.

All patients and donors were classified according to epidemiological risk factors of clinical interest (Table 1). Graft source was unmanipulated mobilized peripheral blood stem cells (PBSCs) in most patients (80 patients, 94.1%). The conditioning regimen for allo-HSCT was myeloablative for 48 patients and reduced intensity conditioning for 37 patients, according to standard clinical practices. GVHD prophylaxis included conventional prophylaxis with Cyclosporine A (CsA) 5 mg/kg per day from day − 1 and Methotrexate (MTX) 15 mg/m2 on day + 1 and 10 mg/m2 on days + 3, + 6, and + 11.

**Table 1:** Patient and donor characteristics and clinical outcomes. Clinical characteristics of the whole cohort and comparison between patients having and lacking CMV reactivation

Parameter	Whole cohort (*n* = 85)	Without CMV reactivation (*n* = 34)	With CMV reactivation (*n* = 51)	*p*-value^a^
Recipient age. median (range), years	44 (13–65)	44 (13–63)	46 (16–63)	0.199
Donor age. median (range), years	44 (11–73)	44 (15–64)	45 (11–73)	0.063
Recipient sex ratio (male/female)	55/30	23/11	32/19	0.817
Donor sex ratio (male/female)	45/40	19/15	25/26	0.658
Diagnosis, no. of patients (%)				
Acute myeloid leukemia	27 (31.8)	12 (35.3)	15 (29.4)	0.638
Non-Hodgkin lymphoma	24 (28.2)	8 (23.5)	16 (31.4)	0.471
Acute lymphoblastic leukemia	16 (18.8)	6 (17.7)	10 (19.5)	1
Myelodysplastic syndrome	8 (9.4)	2 (5.9)	6 (11.8)	0.467
Multiple myeloma	4 (4.7)	3 (8.8)	1 (2.0)	0.297
Hodgkin’s lymphoma	2 (2.4)	1 (2.9)	1 (2.0)	1
Other^b^	4 (4.7)	2 (5.9)	2 (3.9)	1
Stem cell source, *n* (%)				
PB	80 (94.1)	33 (97.1)	47 (92.2)	0.644
Bone marrow	5 (5.9)	1 (2.9)	4 (7.8)	0.644
CMV serostatus, *n* (%)				
D + /R +	60 (70.6)	26 (76.5)	34 (66.7)	0.467
D-/R +	13 (15.3)	3 (8.8)	10 (19.6)	0.227
D + /R-	9 (10.6)	4 (11.8)	5 (9.8)	1
D-/R-	3 (3.5)	1 (2.9)	2 (3.9)	1
Conditioning regimen, *n* (%)				
Myeloablative	48 (56.5)	19 (55.9)	22 (43.1)	1
Reduced-intensity conditioning	37 (43.5)	15 (44.1)	29 (56.9)	1
Prior radiation therapy (TBI), n (%)	15 (17.6)	5 (14.7)	10 (19.6)	0.772
Prior autologous transplant, n (%)	10 (11.8)	4 (11.8)	6 (11.8)	1
aGVHD*, n (%)				
Grade II/IV	47 (55.3)	11 (32.4)	36 (70.6)	**0.001**
Grade III/IV	20 (23.5)	1 (2.9)	19 (37.3)	** < 0.001**

*CMV*, cytomegalovirus; *PB*, peripheral blood; *D*, donor; *R*, recipient; *TBI*, total body irradiation. CMV donor and/or recipient serology represents donor or recipient serological status before transplantation. ^a^Frequency comparisons were performed using the × 2-Fisher exact test. A *P*-value of < 0.05 was considered to be statistically significant. ^b^Other: aplastic anemia, chronic lymphocytic leukemia, chronic myeloid leukemia

### Virological monitoring

Antiviral prophylaxis with 800 mg acyclovir twice daily since admission to 1 year after allo-HSCT was administered to every patient. After allo-HSCT and until day 180 after the infusion, all patients were monitored for CMV reactivation/infection. CMV DNAemia was evaluated twice a week during the first month after allo-HSCT and in a weekly basis thereafter until day 100. CMV assessments were performed on plasma specimen obtained from peripheral blood (PB) using a quantitative real-time PCR assay. CMV reactivation or infection was defined as the detection of CMV DNAemia (threshold level of 100 copies/mL or 155 UI/mL) in one plasma specimen. A new CMV episode was defined as CMV DNAemia detection (> 100 copies/mL) after 2 weeks PCR negativity off antiviral therapy.

### Gene selection

From a predesigned 235-gene panel, we conducted a PubMed search to identify published studies reporting significant associations between one or more human genetic variants and a CMV-related phenotype. A total of 50 genes were selected for their potential role in the pathogenesis of CMV or in any viral infection (Supplementary Table 1). The selected genes correspond to immune-related genes, most of them being cytokines, chemokines and their receptors and some of them have showed association with CVM infection in previous studies.

### Next generation sequencing

Genomic DNA was purified using *Maxwell® RSC Blood DNA Kit* (*Promega*, USA) from 170 PB samples obtained from 85 patients and 85 donors at the pre-transplant evaluation. DNA libraries were performed using a custom enrichment-capture gene panel according to the manufacturer’s protocol (SureSelect, Agilent). Paired-end 2 × 101 bp sequencing was performed using the *HiSeq* platform (*Illumina*, *USA*). FASTQ files were aligned against the human reference genome version GRCh37/hg19 using the Burrows Wheeler Alignment tool v0.7.15-r1140. Variant calling was performed using GATK version 2.8–1, VarScan algorithms and in-house scripts to combine and filter variants. Integrative Genomics Viewer (*Broad Institute*, *USA*) was used to visualize variants aligned against the reference genome to confirm the accuracy of the variant calls by checking for possible strand biases and sequencing errors.

### Variant annotation and filtration

The *Genesystems* software (*Sistemas Genómicos*, Spain) was used for variant annotation, providing the infrastructure and interface for bioinformatic analysis. Transcript annotation of variants was based on all human transcripts obtained from *Ensembl* Released v81. We selected both SNPs and small insertions and deletions (INDELs). Algorithm for variant filtration is described in Supplementary Fig. 1. Variants located in coding regions, in splicing sites of canonical isoforms, and intronic variants were analyzed. Attending to its consequence, synonymous variants were excluded. We selected polymorphisms for which variants had a depth ≥ 30 × and a variant allele frequency (VAF) ≥ 0.4.

In addition, population databases (*GenomAD* and *1000 genomes*) were used to the consult minor allele frequency (MAF) of each variant, in order to identify polymorphisms. We selected variants with MAF greater ≥ 10% in the European population to select polymorphisms that can be applied to routine clinical practice. Finally, *Genecards* and *Uniprot* were used to obtain gene information about the function of the encoded protein, critical domains, etc.

### Statistical analysis

Patient’s characteristics were summarized by means of frequency (n) and percentage (%) for categorical variables and by means of median and range for continuous variables. Differences among groups were evaluated in univariable analysis by the Fisher Exact Test. The Statistical Package for the Social Sciences (SPSS, Chicago, USA) was used for all statistical tests except for cumulative incidence (CI) rates that were calculated using the R Statistical Software (version 3.3.2). Probability values < 0.05 were considered statistically significant.

Multiple logistic regression models were performed with the selected genetic variants that could be applied to clinical practice to anticipate CMV infection and frequency of CMV episodes. Only polymorphisms and clinical variables with *p* < 0.05 in the analysis were included as predictors. The predictive capacity of CMV infection of each model was evaluated using the area under the curve (AUC). Subsequently, the models with the highest AUC value and the lowest number of genetic variants used were selected. To build the risk score, the coefficients of each genetic variant obtained by the logistic model were used. Sensitivity, specificity, positive, and negative predictive values (PPV and NPV) for the different cutoff values were studied. Finally, the score obtained from the chosen predictive model was used to classify patients as low- or high-risk according to the selected cutoff point.

## Results

### Incidence of CMV infection and clinical risk factors

CMV reactivation was observed in 51/85 (60%) patients. In the CMV infection cohort (*n* = 51), 24 patients (47.1%) had only one episode of CMV reactivation, 12 (23.5%) had two different episodes, and 15 patients (29.4%) had more than two episodes within 180 days following allo-HSCT. Initial episodes of CMV reactivation (DNAemia over 100 copies/ml) occurred at a median of 48 days (2–151) after transplant. None of these clinical variants (age, gender, stem cells, hematological disease, conditioning regimen, TBI, prior autologous transplant, and CMV serostatus) were associated with CMV reactivation; only aGVHD was correlated with higher CMV reactivation (Table 1).

Additionally, the relationship between clinical variables with the occurrence of more than 2 CMV reactivations after allo-HSCT was also tested (*n* = 15) Only aGVHD and D/R serology were correlated. Twelve out of fifteen patients with > 2 episodes of CMV reactivation had aGVHD grades II–IV after allo-HSCT, *p* = 0.045; *OR* = 4.0 (1.0–15.4). In addition, 13 cases had D-/R + CMV serology. Six out of thirteen of these patients suffered more than 2 CMV reactivations after allo-HSCT, *p* = 0.009; *OR* = 6.0 (1.6–21.9).

### Variant analysis

Using previously defined bioinformatic filters in our material and methods, 213 SNPs and INDELs were detected in 85 donor-recipient pairs (*n* = 170; Supplementary Table 2, Supplementary Fig. 1). Three variants were specifically detected in recipients (*TGFB1* rs1989457) or donors (*LTA* rs1041981 and rs2229094) and 210 variants were common for both.

### Association between variants in recipient and/or donor and CMV reactivation

**Fig. 1:**
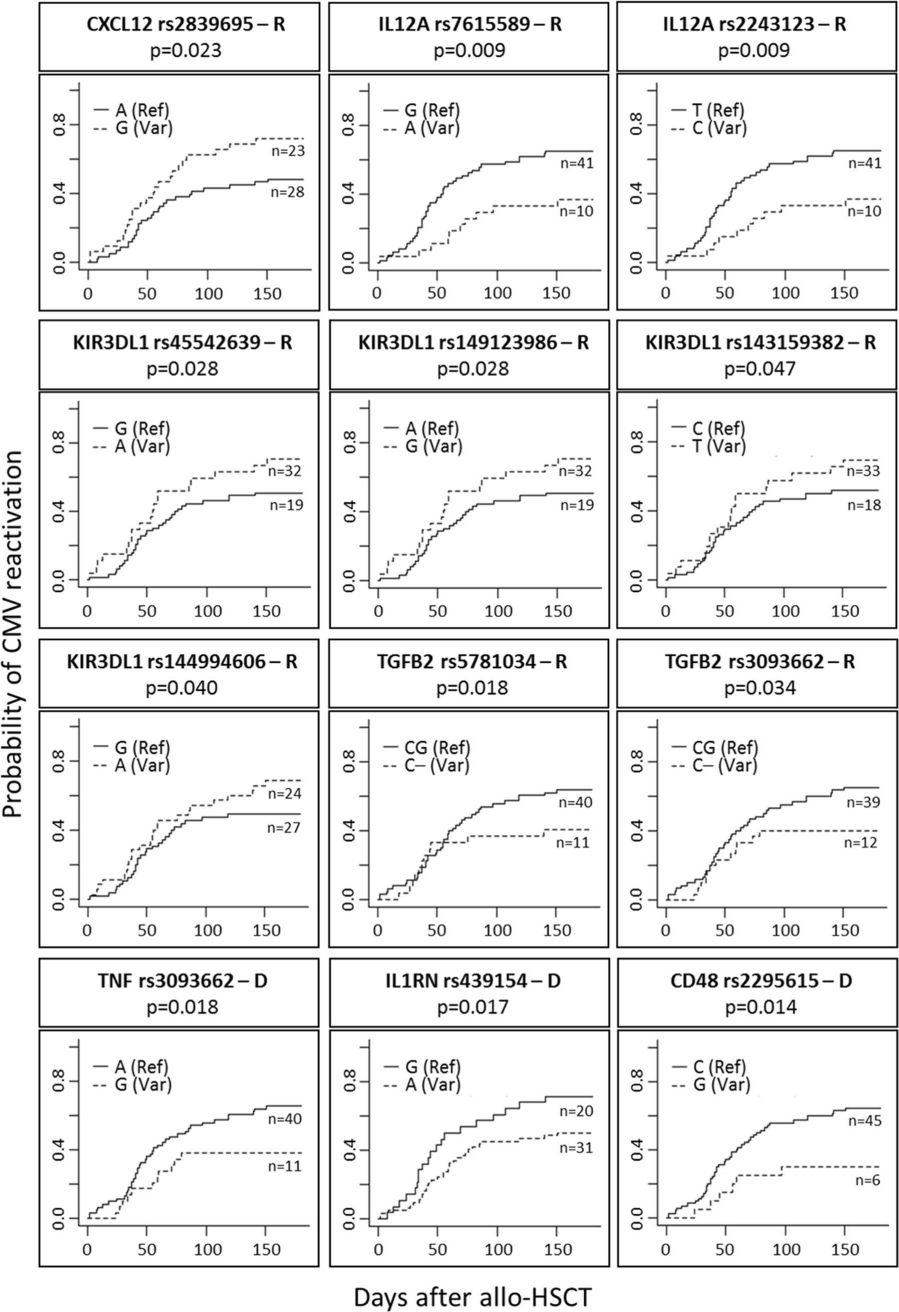
Influence of the genotype of the patient and donor for the polymorphisms selected on the CMV reactivation after allo-HSCT

Genetic analyses for 213 selected SNPs and INDELs were correlated with the development of CMV reactivation in the first 180 days after allo-HSCT. Although 202 variants studied had no apparent impact on CMV reactivation, we found that eleven variants in seven different genes (*CXCL12, IL12A, KIR3DL1, TGFB2, TNF, IL1RN,* and *CD48)* were significantly associated with the risk or protection against CMV reactivation (Table 2, Fig. 1). In the table, we represented variants with *p* < 0.05 (11 variants) and *p*

**Table 2:** Effect of recipient and donor polymorphisms on the incidence rate of CMV reactivation in allo-HSCT patients

CMV reactivation									
Gene	dbSNP number	Genotype (reference/variant)	Recipient/donor	MAF	Variant effect	Yes (*n* = 51)	No (*n* = 34)	*p*-value	Odds ratio (95% *CI*)
*CXCL12*	rs2839695	AA GG/GA	R	0.196	Intron variant (Intron 3/3)	28 23	27 7	0.023*	3.17 (1.17–8.59)
*IL12A*	rs7615589	GG AG/AA	R	0.248	Intron variant (Intron1/6)	41 10	18 16	0.009*	0.27 (0.11–0.72)
	rs2243123	TTCT/CC	R	0.248	Intron variant (Intron 2/6)	4110	1816	0.009*	0.27 (0.11–0.72)
*KIR3DL1*	rs45542639	GGAG/AA	R	0.248	Missense variant(Exon 7/9)	3219	295	0.028*	3.44 (1.14–10.41)
	rs149123986	AAGA/GG	R	0.244	Missense variant(Exon 3/9)	3219	295	0.028*	3.44 (1.14–10.41)
	rs143159382	CCTC/TT	R	0.242	Missense variant(Exon 3/9)	3318	295	0.047	3.16 (1.04–9.59)
	rs144994606	GGAG/AA	R	0.225	Missense variant(Exon 3/9)	2724	268	0.040	2.89 (1.10–7.58)
*TGFB2*	rs5781034	CG C-(delG)	R	0.197	Intron variant (Intron 3/7)	40 11	18 16	0.018*	0.31 (0.12–0.80)
*TNF*	rs3093662	AA GA/GG	R	0.108	Intron variant (Intron 1/3)	39 12	18 16	0.034*	0.35 (0.14–0.88)
	s3093662	AAGA/GG	D	0.108	Intron variant (Intron 1/3)	4011	1816	0.018*	0.31 (0.12–0.80)
*IL1RN*	rs439154	GG AG/AA	D	0.428	Intron variant (Intron 2/5)	20 31	5 29	0.017*	0.27 (0.09–0.81)
*CD48*	rs2295615	CC GC/GG	D	0.105	Missense variant (Exon 2/3)	45 6	22 12	0.014*	0.24 (0.08–0.74)

*R*, recipient; *D*, donor; *MAF*, minor allele frequency. *P < 0.03 (Statistical differences with Bobferroni correction)

### Development of a genetic risk score for CMV reactivation

In order to anticipate CMV reactivation, different predictive models were built using combinations of the 11 polymorphisms selected in the analysis. The parameters used to select the best model were the number of genetic variables and the AUC value (Supplementary Table 3). A model with five genetic polymorphisms (*CXCL12* rs2839695, *IL12A* rs7615589, *KIR3DL1* rs4554639, *TGFB2* rs5781034 for the recipient and *CD48* rs2295615 for the donor; Supplementary Table 4) was selected (AUC: 0.81401, 95% *CI*: 0.71493–0.89020, Supplementary Fig. 2). Score values, odds ratios, and coefficients assigned to each genotype in the prediction model selected are shown in Table 3. The optimal cutoff value to predict the risk of CMV infection, as derived from the analysis of the ROC curves, was defined as 0.49 (Supplementary Table 4). This cutoff value allowed to classify patients as low-risk (< 0.49) or high-risk (≥ 0.49) of suffering CMV infection at pre-transplantation. The sensitivity and specificity of the predictive model were 84.3% (95% *CI*: 72.0–91.8) and 67.6% (95% *CI*

**Table 3:** Polymorphisms included in the CMV predictive model selected. Score values (0 or 1), odds ratio, and coefficient assigned to each genotype in the prediction model

Gene	dbSNP number	R/D	Genotype(Reference/Variant)	Score value	Odds Ratio	95% CI	Coefficient
*CXCL12*	rs2839695	R	AA GG/GA	1 0	3.10	0.97–9.97	1.132
*IL12A*	rs7615589	R	GG AG/AA	0 1	2.49	0.84–7.38	0.914
*KIR3DL1*	rs45542639	R	GG AG/AA	1 0	3.41	0.98–11.84	1.228
*TGFB2*	rs5781034	R	CG C-	0 1	4.33	1.41–13.24	1.465
*CD48*	rs2295615	D	CC GG/GC	0 1	3.13	0.88–11.15	1.140

*R*, recipient; *D*, donor. Score equation calculated according to model selected to applied to each patient: 1/(1 + EXP(-1.132*rs2839695-0.914*rs7615589-1.228*rs4554639-1.465*rs5781034-1.14*rs229561 + 2.733)

**Fig. 2:**
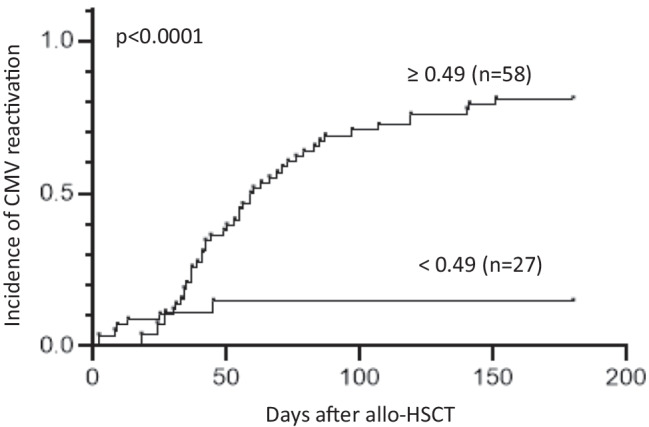
Stratification of the whole cohort of patients according to the risk of CMV reactivation. Risk was calculated using the proposed predictive model which includes five genetic polymorphisms and the cutoff value to predict the risk of CMV infection used was 0.49

Considering that no clinical variables were included in the pre-transplant, polymorphisms based, predictive model constructed, aGVHD grades III–IV were included to re-stratify patients classified as low-risk since this variable was an important risk factor for CMV reactivation in our cohort. Specifically, 4 patients stratified as low-risk of CMV infection (< 0.49) with the pre-transplantation predictive model who suffered grades III–IV aGVHD (Supplementary Table 5) were re-stratified as high-risk. Then, we calculated the CMV risk score for each patient (defined by score value ≥ 0.49 or grade III/IV GVHD) to test the usefulness of the model to identify patients at high-risk of experiencing a CMV reactivation after allo-HSCT. Out of the 85 participants, 58 (68.2%) were classified as high-risk and 27 (31.8%) as low-risk. At 100 days after allo-HSCT, 70.7% of patients with a high-risk score experienced CMV reactivation compared to 14.8% of those with a low-risk score (Fig. 2). Therefore, the CMV predictive score was able to correctly stratify patients according to their risk of developing CMV reactivation (*p*

We also calculated the CMV risk score for only R + patient. Out of the 73 patients, 46 were classified as high risk and 27 as low risk. At 180 days after allo-HSCT, 78.2% of patients with a high-risk score experienced CMV reactivation compared to 29.6% of those with a low-risk score. Therefore, the CMV predictive score was able to correctly stratify patients according to their risk of developing CMV reactivation in R + cohort.

## Discussion

CMV remains as one of the most common clinically significant viral infections after allo-HSCT. Over the past decade, most centers have adopted a preemptive strategy in which CMV surveillance and detection in blood by different methods trigger antiviral treatment to prevent clinical CMV disease and minimize the toxic effects of these antiviral agents [18]. In recent years, gene-polymorphism studies have shown clinical utility in different settings. Polymorphisms in certain cytokine and chemokine genes showed significant association with the presentation of viral infections in allo-HSCT recipients, essentially with CMV infection [19–23]. Recently, Casto A et al. suggested that the G allele for rs1045642 in *ABCB1* in donors reduces the risk of CMV reactivation by approximately 20%, because these are related with lower intracellular calcineurin inhibitor concentrations [24].

Our data suggest a significantly lower risk of CMV reactivation in recipients and donors with particular cytokine loci variants. In particular, our data showed that the *TNF*-α SNP rs3093662 (GA/GG) genotype in donors and recipients was associated with protection from CMV infection after allo-HSCT. The presence of allele G in this position has been associated with an increase in transcriptional activity and high in vitro production of TNF-α [25]. CMV is a potent inducer of TNF-α production but this cytokine has direct antiviral effects and, together with IL1, potentiates the lytic activity of NK cells [26]. On other hand, IL12 plays a crucial role in anti-infectious immune responses, especially by stimulating IFNγ production [27]. Hoffmann TW et al. investigated the 3´UTR polymorphism (rs3212227) of the IL12p40 gene and shown that the C allele is associated with lower production of the p40 polypeptide, which might explain the pathogenic link between the *IL12B* genotype and CMV replication [28]. In contrast to Hoffmann et al., we noted an association between the *IL12A* polymorphisms rs7615589 and rs2243123 in the recipient and protection against CMV infection. Although *TGFB1* alleles were found to be significantly associated with CMV reactivation, our data also suggest a significantly lower risk of CMV reactivation in patients with *TGFB2* rs5781034 SNP. This gene encodes a secreted ligand of the TGF-beta superfamily of proteins. TGFβ-2 protein helps control the growth, differentiation and proliferation of cells.

On the other hand, we demonstrated that *CD48* rs2295615 (GC/GG) and *IL1RN* rs439154 (AG/AA) in the donor was associated with protection for CMV reactivation. However, the exact mechanism by which these SNPs exert their activity is not well established. These could culminate in trans-activation of a repertoire of pro-inflammatory cytokines that would favor the elimination of the infectious agents. Individuals homozygous for specific alleles for the *IL1RN* gene present a high prolonged and severe proinflammatory immune response [29]. In our cohort, *IL1RN* rs439154 (AG/AA) in the donor was also associated with protection from CMV reactivation. Hurme M et al. have shown that the number of 86-bp tandem repeats in intron 2 of the *IL1RN* gene are associated with an increased inflammatory response provoked by an infectious disease [30]. In conclusion, our data suggested that particular polymorphisms in cytokine genes such as *TNF-α*, *LTA*, *IL12*, *TGBF2*, *and IL1RN* could play an important role in increasing pro-inflammatory cytokine expression in T cells and NK cells, which might be beneficial in protection against CMV infection. On the other hand, our data showed that the *CXCL12* rs2839695 (GG/AG) SNP in the recipient was associated with an increased risk of CMV infection. An increased expression of *CXCL12* would promote local inflammatory responses that trigger CMV reactivation and induce CMV replication. *CXCL12* signaling was amplified in CMV-infected cells [31].

Most of the *KIR* genes exhibit allelic polymorphism and this serves to diversify *KIR* haplotypes, which define different levels of cell surface expression. Several authors reported associations between the expression of *KIR* genes in allo-HSCT donors and patients and the risk of CMV infection [32, 33]. De Rham et al. explored the expression of *KIR3DL1* on NK cells during acute CMV infection and showed high levels of expression, which implies that this receptor could be involved in clearing CMV infection [34]. Our analysis showed a significantly higher risk of CMV reactivation in recipients with *KIR3DL1* rs45542639 (AG/AA), rs149123986 (GA/GG), rs144994606 (AG/AA), and rs143159382 (TC/TT). However, the exact mechanism by which these SNPs influence KIR3DL1 expression is not well established, so an extensive study would better elucidate whether these *KIR3DL1* genetic variants may modify the risk of CMV infections after allo-HSCT.

Finally, with the objective of increasing the clinical utility of our results, we built the best-possible predictive model with our data to improve the prediction of CMV reactivation after allo-HSCT. Our results suggest that particular SNPs in recipient and donors (pre-transplantation model), in combination with the development of grades III-IV aGVHD, could predict CMV reactivation/infection. The model proposed can be readily applied by other centers using the predictive score built in this study.

Regarding clinical variables, important risk factors for CMV infections are the serological status of donor and recipient, aGVHD, and T-cell depletion. It has been established that CMV seropositive patients show a significantly higher incidence of CMV infection than CMV-seronegative recipients [35]. Our study is unique in that CMV serologic status did not influence the rate of CMV infection. The presented cohort has 85% of R + patients, so only a minority of patients was CMV-seronegative recipients. It is possible that the impact of CMV serostatus will vary by increasing the number of R- and, consequently, CMV serostatus may include in the CMV predictive score. In our cohort, CMV D-/R + serologic status only influenced the occurrence of more than two CMV reactivation episodes. According to other authors, we also confirmed a strong association between aGVHD (grades II/IV and III/IV) and CMV infections [36].

To our knowledge, this is the first preliminary report contributing a pre-transplant genetic risk score useful to identify patients at high-risk of experiencing a CMV reactivation after allo-HSCT. This approach could facilitate personalized risk-adapted clinical management of patients undergoing allo-HSCT. Nevertheless, the study reported in this manuscript is exploratory and cannot be considered complete until further work is done to verify the performance of the model in an independent cohort. As it stands, the predictive model was optimized to fit the observed data in a relatively small cohort. Therefore, the results should be considered preliminary and an external validation is needed. In addition, these polymorphisms should be validated in a larger cohort and in other hematopoietic stem cell transplant settings such as the currently widely used haploidentical stem cell transplantation with post-transplant cyclophosphamide (PT/Cy) based GVHD prophylaxis. Retrospective studies of allo-HSCT with PT/Cy have shown a high incidence of CMV viremia and large collaborative studies in PT/Cy patients would elucidate whether genetic variants impact viral infections in this setting [37].

In summary, the data presented here suggest that screening of patients and donors’ pre-transplantation helps to predict the individual risk of the development of CMV infection and disease after an HLA-identical allo-HSCT. These results might also allow the identification of patients at high-risk of CMV reactivation after transplantation, enabling pre-emptive therapy or attempts to cure the infection by administering antiviral therapy or CMV-specific T lymphocytes.

## Supplementary Information

Supplementary file1 (DOCX 149 KB)
