# Supplementary material for: Risk prediction of CMV reactivation after allogeneic stem cell transplantation using five non-HLA immunogenetic polymorphisms
Source: Ann Hematol. 2022 May 7;101(7):1567–76. doi: 10.1007/s00277-022-04841-8 (PMC9203380; doi:10.1007/s00277-022-04841-8)
Supplement: Supplementary file 1 — Supplementary file1 (DOCX 149 KB) [file 277_2022_4841_MOESM1_ESM.docx]

**SUPPLEMENTARY MATERIAL**

**SUPPLEMENTARY FIGURES: 1-2**

**Total variants (R and D)**

Synonymous variants excluded

Depth ≥ 30

Canonical Isoforms

**Coding and splicing**

(1) R/D: 271

(2) R: 39

(3) D: 40

**Intronic**

**Supplementary figure 1.** Bioinformatic pipeline to identify SNPs and INDELs in patients and donors.

D: donor; R: recipient; VAF: variant allele frequency; MAF**:** minor allele frequency.

(1) R/D: common variants for both R and D; (2) R: variants present in the R; (3) D: variants present in the D.

**VAF ≥ 0.4**

(1) R/D: 196

(2) R: 34

(3) D: 39

**VAF ≥ 0.4**

(1) R/D: 405

(2) R: 62

(3) D: 57

**MAF ≥ 10%**

69 for R/D

0 for R

2 for D

**MAF ≥ 10%**

141 for R/D

1 for R

0 for D

**Supplementary figure 2**. Analysis of receiver operator characteristic (ROC) curve for predictive models selected, m5 and m6.

**SUPPLEMENTARY MATERIAL: TABLES 1-5**

**Supplementary table 1**. Immune related genes selected for the study due to their potential role in the pathogenesis of viral infections.

| **Gene Symbol** | **Gene Id** | **Gene Description** | **References** |
| --- | --- | --- | --- |
| *CCL4* | 10630 | C-C motif chemokine ligand 4 |  |
| *CCL5* | 10632 | C-C motif chemokine ligand 5 | [1] |
| *CCL8* | 10635 | C-C motif chemokine ligand 8 | [2] |
| *CCR5* | 1606 | [C-C motif chemokine receptor 5](https://www.genenames.org/data/gene-symbol-report/#!/hgnc_id/HGNC:1606) | [3–5] |
| *CCR8* | 1609 | C-C motif chemokine receptor 8 |  |
| *CD48* | 1683 | CD48 molecule |  |
| *CXCL1* | 2919 | C-X-C motif chemokine ligand 1 | [6] |
| *CXCL10* | 3627 | C-X-C motif chemokine ligand 10 | [7] |
| *CXCL12* | 10672 | C-X-C motif chemokine ligand 12 | [8] |
| *CXCR2* | 6027 | C-X-C motif chemokine receptor 2 | [6] |
| *CXCR4* | 2561 | C-X-C motif chemokine receptor 4 | [8] |
| *FOXP3* | 6106 | Forkhead box P3 | [9] |
| *IFNAR1* | 5432 | interferon alpha and beta receptor subunit 1 |  |
| *IFNAR2* | 5433 | interferon alpha and beta receptor subunit 2 |  |
| *IFNG* | 5438 | Interferon gamma | [10–13] |
| *IFNGR1* | 5439 | interferon gamma receptor 1 |  |
| *IFNGR2* | 5440 | interferon gamma receptor 2 |  |
| *IFNK* | 21714 | Interferon kappa |  |
| *IFNL1* | 18363 | Interferon lambda 1 |  |
| *IFNL2* | 18364 | Interferon lambda 2 |  |
| *IFNL3* | 18365 | Interferon lambda 3 |  |
| *IL10* | 5962 | interleukin 10 | [3, 4, 11, 14] |
| *IL10RA* | 5964 | interleukin 10 receptor subunit alpha |  |
| *IL10RB* | 5965 | interleukin 10 receptor subunit beta |  |
| *IL12A* | 5969 | interleukin 12A | [15] |
| *IL12RB1* | 5971 | interleukin 12 receptor subunit beta 1 |  |
| *IL12RB2* | 5972 | interleukin 12 receptor subunit beta 2 |  |
| *IL1A* | 5991 | interleukin 1 alpha | [16, 17] |
| *IL1B* | 5992 | interleukin 1 beta | [16, 17] |
| *IL1RL1* | 5998 | interleukin 1 receptor like 1 |  |
| *IL1RL2* | 5999 | interleukin 1 receptor like 2 |  |
| *IL1RN* | 6000 | interleukin 1 receptor antagonist | [18] |
| *IL2* | 6001 | interleukin 2 | [19] |
| *IL2RA* | 6008 | interleukin 2 receptor subunit alpha |  |
| *IL2RB* | 6009 | interleukin 2 receptor subunit beta |  |
| *IL4* | 6014 | interleukin 4 | [19, 20] |
| *IL4R* | 6015 | interleukin 4 receptor | [20] |
| *IL6* | 6018 | interleukin 6 | [10, 11, 16] |
| *IL6R* | 6019 | interleukin 6 receptor |  |
| *IL7R* | 6024 | interleukin 7 receptor | [21] |
| *KIR2DL1* | 6329 | killer cell immunoglobulin-like receptor, two domains, long cytoplasmic tail, 1 |  |
| *KIR2DL3* | 6331 | killer cell immunoglobulin-like receptor, two domains, long cytoplasmic tail, 3 |  |
| *KIR2DS4* | 6336 | killer cell immunoglobulin-like receptor, two domains, short cytoplasmic tail, 4 | [22, 23] |
| *KIR3DL1* | 6338 | killer cell immunoglobulin-like receptor, three domains, long cytoplasmic tail, 1 | [24] |
| *KIR3DL2* | 6339 | killer cell immunoglobulin-like receptor, three domains, long cytoplasmic tail, 2 |  |
| *KIR3DL3* | 16312 | killer cell immunoglobulin-like receptor, three domains, long cytoplasmic tail, 3 |  |
| *LTA* | 6709 | lymphotoxin alpha |  |
| *TGFB1* | 11766 | transforming growth factor beta 1 | [19] |
| *TGFB2* | 11768 | transforming growth factor beta 2 | [19] |
| *TNF* | 11892 | tumor necrosis factor | [10, 18, 25] |

**References for Supplementary table 1.**

1. Kim DH, Jung H Du, Lee NY, Sohn SK (2007) Single nucleotide polymorphism of CC chemokine ligand 5 promoter gene in recipients may predict the risk of chronic graft-versus-host disease and its severity after allogeneic transplantation. Transplantation 84:917–25. https://doi.org/10.1097/01.tp.0000284583.15810.6e

2. Lisboa LF, Egli A, Fairbanks J, et al (2015) CCL8 and the Immune Control of Cytomegalovirus in Organ Transplant Recipients. American journal of transplantation : official journal of the American Society of Transplantation and the American Society of Transplant Surgeons 15:1882–92. https://doi.org/10.1111/ajt.13207

3. Corrales I, Giménez E, Solano C, et al (2015) Incidence and dynamics of active cytomegalovirus infection in allogeneic stem cell transplant patients according to single nucleotide polymorphisms in donor and recipient CCR5, MCP-1, IL-10, and TLR9 genes. Journal of medical virology 87:248–55. https://doi.org/10.1002/jmv.24050

4. Loeffler J, Steffens M, Arlt E-M, et al (2006) Polymorphisms in the genes encoding chemokine receptor 5, interleukin-10, and monocyte chemoattractant protein 1 contribute to cytomegalovirus reactivation and disease after allogeneic stem cell transplantation. Journal of clinical microbiology 44:1847–50. https://doi.org/10.1128/JCM.44.5.1847-1850.2006

5. Bogunia-Kubik K, Jaskula E, Lange A (2007) The presence of functional CCR5 and EBV reactivation after allogeneic haematopoietic stem cell transplantation. Bone marrow transplantation 40:145–50. https://doi.org/10.1038/sj.bmt.1705703

6. Heo J, Dogra P, Masi TJ, et al (2015) Novel Human Cytomegalovirus Viral Chemokines, vCXCL-1s, Display Functional Selectivity for Neutrophil Signaling and Function. The Journal of Immunology 195:227–236. https://doi.org/10.4049/jimmunol.1400291

7. Uhlin M, Mattsson J, Maeurer M (2012) Update on viral infections in lung transplantation. Current Opinion in Pulmonary Medicine 18:264–270. https://doi.org/10.1097/MCP.0b013e3283521066

8. Tu CC, Arnolds KL, O’connor CM, Spencer J V (2018) Human Cytomegalovirus UL111A and US27 Gene Products Enhance the CXCL12/CXCR4 Signaling Axis via Distinct Mechanisms. https://doi.org/10.1128/JVI.01981-17

9. Piao Z, Kim HJ, Choi JY, et al (2016) Effect of FOXP3 polymorphism on the clinical outcomes after allogeneic hematopoietic stem cell transplantation in pediatric acute leukemia patients. International immunopharmacology 31:132–9. https://doi.org/10.1016/j.intimp.2015.12.022

10. Mitsani D, Nguyen MH, Girnita DM, et al (2011) A polymorphism linked to elevated levels of interferon-γ is associated with an increased risk of cytomegalovirus disease among Caucasian lung transplant recipients at a single center. The Journal of heart and lung transplantation : the official publication of the International Society for Heart Transplantation 30:523–9. https://doi.org/10.1016/j.healun.2010.11.008

11. Alakulppi NS, Kyllönen LE, Salo HME, et al (2006) The impact of donor cytokine gene polymorphisms on the incidence of cytomegalovirus infection after kidney transplantation. Transplant Immunology 16:258–262. https://doi.org/10.1016/j.trim.2006.09.007

12. Cai X, Song A, Wang H, et al (2012) [Role of IFN-γ + 874 genetic polymorphisms in allogeneic hematopoietic stem cell transplantation]. Zhonghua xue ye xue za zhi = Zhonghua xueyexue zazhi 33:989–93

13. Vu D, Shah T, Ansari J, et al (2014) Interferon-gamma gene polymorphism +874 A/T is associated with an increased risk of cytomegalovirus infection among Hispanic renal transplant recipients. Transplant infectious disease : an official journal of the Transplantation Society 16:724–32. https://doi.org/10.1111/tid.12285

14. Vu D, Shah T, Ansari J, et al (2014) Interferon-gamma gene polymorphism +874 A/T is associated with an increased risk of cytomegalovirus infection among Hispanic renal transplant recipients. Transplant Infectious Disease 16:724–732. https://doi.org/10.1111/tid.12285

15. Hoffmann TW, Halimi J-M, Büchler M, et al (2008) Association Between a Polymorphism in the IL-12p40 Gene and Cytomegalovirus Reactivation After Kidney Transplantation. Transplantation 85:1406–1411. https://doi.org/10.1097/TP.0b013e31816c7dc7

16. Wujcicka WI, Wilczyński JS, Nowakowska DE (2017) Association of SNPs from *IL1A* , *IL1B* , and *IL6* Genes with Human Cytomegalovirus Infection Among Pregnant Women. Viral Immunology 30:288–297. https://doi.org/10.1089/vim.2016.0129

17. Wujcicka W, Wilczyński J, Paradowska E, et al (2017) The role of single nucleotide polymorphisms, contained in proinflammatory cytokine genes, in the development of congenital infection with human cytomegalovirus in fetuses and neonates. Microbial pathogenesis 105:106–116. https://doi.org/10.1016/j.micpath.2017.02.017

18. Hurme M, Helminen M (1998) Resistance to human cytomegalovirus infection may be influenced by genetic polymorphisms of the tumour necrosis factor-alpha and interleukin-1 receptor antagonist genes. Scandinavian journal of infectious diseases 30:447–9

19. Cano P, Han FS, Wang H-L, et al (2012) Cytokine gene polymorphisms affect reactivation of cytomegalovirus in patients with cancer. Cytokine 60:417–422. https://doi.org/10.1016/j.cyto.2012.07.018

20. Lee HJ, Kim TH, Kang SW, et al (2016) Association Interleukin-4 and Interleukin-4 Receptor Gene Polymorphism and Acute Rejection and Graft Dysfunction After Kidney Transplantation. Transplantation proceedings 48:813–9. https://doi.org/10.1016/j.transproceed.2015.12.059

21. Kielsen K, Enevold C, Heilmann C, et al (2018) Donor Genotype in the Interleukin-7 Receptor α-Chain Predicts Risk of Graft-versus-Host Disease and Cytomegalovirus Infection after Allogeneic Hematopoietic Stem Cell Transplantation. Frontiers in immunology 9:109. https://doi.org/10.3389/fimmu.2018.00109

22. Gallez-Hawkins GM, Franck AE, Li X, et al (2011) Expression of Activating KIR2DS2 and KIR2DS4 Genes after Hematopoietic Cell Transplantation: Relevance to Cytomegalovirus Infection. Biology of Blood and Marrow Transplantation 17:1662–1672. https://doi.org/10.1016/j.bbmt.2011.04.008

23. Wu X, Yao Y, Bao X, et al (2016) KIR2DS4 and Its Variant KIR1D Are Associated with Acute Graft-versus-Host Disease, Cytomegalovirus, and Overall Survival after Sibling-Related HLA-Matched Transplantation in Patients with Donors with KIR Gene Haplotype A. Biology of blood and marrow transplantation : journal of the American Society for Blood and Marrow Transplantation 22:220–225. https://doi.org/10.1016/j.bbmt.2015.10.004

24. Chen C, Busson M, Rocha V, et al (2006) Activating KIR genes are associated with CMV reactivation and survival after non-T-cell depleted HLA-identical sibling bone marrow transplantation for malignant disorders. Bone marrow transplantation 38:437–44. https://doi.org/10.1038/sj.bmt.1705468

25. Arav-Boger R, Willoughby RE, Pass RF, et al (2002) Polymorphisms of the cytomegalovirus (CMV)-encoded tumor necrosis factor-alpha and beta-chemokine receptors in congenital CMV disease. The Journal of infectious diseases 186:1057–64. https://doi.org/10.1086/344238

**Supplementary table 2**. Polymorphisms (n=213) detected in 85 donor-recipient pairs using filters defined previously.

| **R/D** | **Gene** | **Chr** | **Position** | **Reference** | **Variant** | **HGVSc** | **HGVSp** | **Variant Effect** | **dbSNP number** | **MAF** |
| --- | --- | --- | --- | --- | --- | --- | --- | --- | --- | --- |
| R/D | CCL4 | 17 | 36105271 | T | A | ENST00000615863.1:c.238T>A | ENSP00000482259.1:p.Ser80Thr | missense variant | rs1719152 | 0.249 |
| R/D | CCL5 | 17 | 35879999 | A | G | ENST00000603197.5:c.76+231T>C |  | intron variant | rs2280789 | 0.113 |
| R/D | CCL8 | 17 | 34320812 | A | C | ENST00000394620.1:c.205A>C | ENSP00000378118.1:p.Lys69Gln | missense variant | rs1133763 | 0.151 |
| R/D | CCR5 | 3 | 46373452 | TACAGTCAGTATCAATTCTGGAAGAATTTCCAG | T | ENST00000292303.4:c.554_585delGTCAGTATCAATTCTGGAAGAATTTCCAGACA | ENSP00000292303.4:p.Ser185IlefsTer32 | frameshift variant | rs562091107 | 0.110 |
| R/D | CCR8 | 3 | 39332411 | C | G | ENST00000326306.4:c.80C>G | ENSP00000326432.4:p.Ala27Gly | missense variant | rs2853699 | 0.298 |
| R/D | CD48 | 1 | 160684968 | C | G | ENST00000613788.1:c.304G>C | ENSP00000484431.1:p.Glu102Gln | missense variant | rs2295615 | 0.105 |
| R/D | CXCL12 | 10 | 44382053 | T | C | ENST00000395794.2:c.62-1173A>G |  | intron variant | rs11592974 | 0.191 |
| R/D | CXCL12 | 10 | 44382666 | A | G | ENST00000395794.2:c.62-1786T>C |  | intron variant | rs17879548 | 0.101 |
| R/D | CXCL12 | 10 | 44383610 | G | T | ENST00000395794.2:c.61+1335C>A |  | intron variant | rs191790682 | 0.101 |
| R/D | CXCL12 | 10 | 44380434 | C | G | ENST00000395794.2:c.179+329G>C |  | intron variant | rs2839688 | 0.109 |
| R/D | CXCL12 | 10 | 44378544 | C | CCT | ENST00000395794.2:c.266+91_266+92dupAG |  | intron variant | rs2839694 | 0.259 |
| R/D | CXCL12 | 10 | 44378401 | A | G | ENST00000395794.2:c.266+236T>C |  | intron variant | rs2839695 | 0.196 |
| R/D | CXCL12 | 10 | 44383265 | G | A | ENST00000395794.2:c.61+1680C>T |  | intron variant | rs3780891 | 0.101 |
| R/D | CXCL12 | 10 | 44383193 | T | C | ENST00000395794.2:c.61+1752A>G |  | intron variant | rs7088876 | 0.196 |
| R/D | CXCL12 | 10 | 44383384 | T | C | ENST00000395794.2:c.61+1561A>G |  | intron variant | rs7092453 | 0.198 |
| R/D | CXCL12 | 10 | 44384263 | A | G | ENST00000395794.2:c.61+682T>C |  | intron variant | rs7093481 | 0.297 |
| R/D | CXCL12 | 10 | 44384759 | C | G | ENST00000395794.2:c.61+186G>C |  | intron variant | rs72790861 | 0.297 |
| R/D | CXCL12 | 10 | 44384812 | T | C | ENST00000395794.2:c.61+133A>G |  | intron variant | rs72790862 | 0.297 |
| R/D | CXCL12 | 10 | 44383518 | C | T | ENST00000395794.2:c.61+1427G>A |  | intron variant | rs74427555 | 0.101 |
| R/D | CXCL12 | 10 | 44384833 | C | T | ENST00000395794.2:c.61+112G>A |  | intron variant | rs78453259 | 0.101 |
| R/D | FOXP3 | X | 49259429 | T | C | ENST00000376207.8:c.-22-902A>G |  | intron variant | rs2232365 | 0.456 |
| R/D | FOXP3 | X | 49252667 | T | C | ENST00000376207.8:c.1044+459A>G |  | intron variant | rs2280883 | 0.350 |
| R/D | IFNAR1 | 21 | 33343393 | G | C | ENST00000270139.7:c.502G>C | ENSP00000270139.3:p.Val168Leu | missense variant | rs2257167 | 0.129 |
| R/D | IFNAR2 | 21 | 33241950 | T | G | ENST00000342136.8:c.28T>G | ENSP00000343957.4:p.Phe10Val | missense variant | rs1051393 | 0.333 |
| R/D | IFNG | 12 | 68157629 | T | C | ENST00000229135.3:c.366+284G>A |  | intron variant | rs1861494 | 0.272 |
| R/D | IFNG | 12 | 68158742 | T | A | ENST00000229135.3:c.115-483A>T |  | intron variant | rs2430561 | 0.462 |
| R/D | IFNGR1 | 6 | 137198643 | T | C | ENST00000367739.8:c.862-4A>G |  | splice region variant | rs3799488 | 0.130 |
| R/D | IFNGR2 | 21 | 33415005 | A | G | ENST00000381995.5:c.248A>G | ENSP00000371425.1:p.Gln83Arg | missense variant | rs9808753 | 0.143 |
| R/D | IFNL1 | 19 | 39298475 | A | G | ENST00000333625.2:c.562A>G | ENSP00000329991.1:p.Asn188Asp | missense variant | rs30461 | 0.112 |
| R/D | IFNL2 | 19 | 39269795 | T | C | ENST00000331982.5:c.478T>C | ENSP00000333639.4:p.Tyr160His | missense variant | rs59746524 | 0.208 |
| R/D | IFNL2 | 19 | 39269551 | A | G | ENST00000331982.5:c.334A>G | ENSP00000333639.4:p.Thr112Ala | missense variant | rs8103362 | 0.226 |
| R/D | IFNL3 | 19 | 39244466 | T | C | ENST00000413851.2:c.209A>G | ENSP00000409000.2:p.Lys70Arg | missense variant | rs8103142 | 0.312 |
| R/D | IL10 | 1 | 206771300 | C | T | ENST00000423557.1:c.225+56A>G |  | intron variant | rs1518111 | 0.221 |
| R/D | IL10 | 1 | 206770368 | G | C | ENST00000423557.1:c.379-474C>G |  | intron variant | rs1878672 | 0.453 |
| R/D | IL10 | 1 | 206770767 | T | A | ENST00000423557.1:c.378+140A>T |  | intron variant | rs3024492 | 0.222 |
| R/D | IL10 | 1 | 206770623 | C | A | ENST00000423557.1:c.378+284G>T |  | intron variant | rs3024493 | 0.166 |
| R/D | IL10 | 1 | 206769068 | C | T | ENST00000423557.1:c.445-340G>A |  | intron variant | rs3024495 | 0.168 |
| R/D | IL10RA | 11 | 117998955 | G | A | ENST00000227752.7:c.1051A>G | ENSP00000227752.3:p.Arg351Gly | missense variant | rs2229113 | 0.318 |
| R/D | IL10RA | 11 | 117993348 | A | G | ENST00000227752.7:c.475A>G | ENSP00000227752.3:p.Ser159Gly | missense variant | rs3135932 | 0.167 |
| R/D | IL10RB | 21 | 33268483 | A | G | ENST00000290200.6:c.139A>G | ENSP00000290200.2:p.Lys47Glu | missense variant | rs2834167 | 0.268 |
| R/D | IL12A | 3 | 159994267 | T | G | ENST00000305579.6:c.606+423G>T |  | intron variant | rs2227314 | 0.468 |
| R/D | IL12A | 3 | 159991864 | T | C | ENST00000305579.6:c.265-1148T>C |  | intron variant | rs2243123 | 0.248 |
| R/D | IL12A | 3 | 159994271 | A | C | ENST00000305579.6:c.606+427A>C |  | intron variant | rs2243131 | 0.149 |
| R/D | IL12A | 3 | 159995206 | G | C | ENST00000305579.6:c.607-198G>C |  | intron variant | rs2243135 | 0.383 |
| R/D | IL12A | 3 | 159995300 | T | C | ENST00000305579.6:c.607-104T>C |  | intron variant | rs2243136 | 0.133 |
| R/D | IL12A | 3 | 159992541 | G | C | ENST00000305579.6:c.265-471G>C |  | intron variant | rs475825 | 0.149 |
| R/D | IL12A | 3 | 159992214 | T | A | ENST00000305579.6:c.265-798A>T |  | intron variant | rs582054 | 0.468 |
| R/D | IL12A | 3 | 159992311 | C | A | ENST00000305579.6:c.265-701A>C |  | intron variant | rs582537 | 0.468 |
| R/D | IL12A | 3 | 159989399 | G | A | ENST00000305579.6:c.118+225G>A |  | intron variant | rs7615589 | 0.248 |
| R/D | IL12RB1 | 19 | 18077598 | C | T | ENST00000600835.6:c.467G>A | ENSP00000470788.1:p.Arg156His | missense variant | rs11575926 | 0.155 |
| R/D | IL12RB1 | 19 | 18075808 | T | C | ENST00000600835.6:c.641A>G | ENSP00000470788.1:p.Gln214Arg | missense variant | rs11575934 | 0.311 |
| R/D | IL12RB1 | 19 | 18069641 | A | G | ENST00000600835.6:c.1094T>C | ENSP00000470788.1:p.Met365Thr | missense variant | rs375947 | 0.313 |
| R/D | IL12RB1 | 19 | 18069603 | C | G | ENST00000600835.6:c.1132G>C | ENSP00000470788.1:p.Gly378Arg | missense variant | rs401502 | 0.312 |
| R/D | IL12RB1 | 19 | 18086934 | T | A | ENST00000600835.6:c.-109-2A>T |  | splice acceptor variant | rs393548 | 0.198 |
| R/D | IL1A | 2 | 112781205 | A | G | ENST00000263339.3:c.319+399C>T |  | intron variant | rs1533463 | 0.320 |
| R/D | IL1A | 2 | 112782628 | T | G | ENST00000263339.3:c.96+88C>A |  | intron variant | rs1609682 | 0.322 |
| R/D | IL1A | 2 | 112782600 | C | T | ENST00000263339.3:c.96+116G>A |  | intron variant | rs1894399 | 0.287 |
| R/D | IL1A | 2 | 112782507 | A | G | ENST00000263339.3:c.96+209C>T |  | intron variant | rs2071373 | 0.321 |
| R/D | IL1A | 2 | 112779775 | T | G | ENST00000263339.3:c.320-109A>C |  | intron variant | rs2071374 | 0.268 |
| R/D | IL1A | 2 | 112777861 | C | T | ENST00000263339.3:c.615+126G>A |  | intron variant | rs2071375 | 0.287 |
| R/D | IL1A | 2 | 112777818 | T | G | ENST00000263339.3:c.615+169C>A |  | intron variant | rs2071376 | 0.322 |
| R/D | IL1A | 2 | 112784348 | G | A | ENST00000263339.3:c.-9+95C>T |  | intron variant | rs2856837 | 0.287 |
| R/D | IL1A | 2 | 112782395 | G | A | ENST00000263339.3:c.96+321C>T |  | intron variant | rs2856838 | 0.391 |
| R/D | IL1A | 2 | 112779762 | A | G | ENST00000263339.3:c.320-96T>C |  | intron variant | rs2856841 | 0.287 |
| R/D | IL1A | 2 | 112784242 | A | T | ENST00000263339.3:c.-9+201A>T |  | intron variant | rs3783525 | 0.322 |
| R/D | IL1A | 2 | 112784230 | C | T | ENST00000263339.3:c.-9+213A>G |  | intron variant | rs3783526 | 0.321 |
| R/D | IL1A | 2 | 112781202 | A | G | ENST00000263339.3:c.319+402C>T |  | intron variant | rs3783533 | 0.321 |
| R/D | IL1A | 2 | 112779074 | G | A | ENST00000263339.3:c.490+422T>C |  | intron variant | rs3783543 | 0.322 |
| R/D | IL1A | 2 | 112777253 | C | G | ENST00000263339.3:c.615+734C>G |  | intron variant | rs3783546 | 0.322 |
| R/D | IL1A | 2 | 112775308 | T | G | ENST00000263339.3:c.616-41C>A |  | intron variant | rs3783550 | 0.322 |
| R/D | IL1A | 2 | 112779646 | C | A | ENST00000263339.3:c.340G>T | ENSP00000263339.3:p.Ala114Ser | missense variant | rs17561 | 0.287 |
| R/D | IL1B | 2 | 112831756 | C | T | ENST00000263341.6:c.467-334G>A |  | intron variant | rs1143637 | 0.245 |
| R/D | IL1B | 2 | 112831216 | C | T | ENST00000263341.6:c.597+76G>A |  | intron variant | rs1143639 | 0.245 |
| R/D | IL1B | 2 | 112831178 | CAA | C | ENST00000263341.6:c.597+112_597+113delTT |  | intron variant | rs1143640 | 0.245 |
| R/D | IL1B | 2 | 112830725 | C | T | ENST00000263341.6:c.598-152G>A |  | intron variant | rs1143643 | 0.338 |
| R/D | IL1B | 2 | 112835315 | GA | G | ENST00000263341.6:c.99+250delT |  | intron variant | rs3917354 | 0.204 |
| R/D | IL1B | 2 | 112834786 | C | T | ENST00000263341.6:c.99+780G>A |  | intron variant | rs3917356 | 0.436 |
| R/D | IL1RL1 | 2 | 102351751 | C | A | ENST00000233954.5:c.1501C>A | ENSP00000233954.1:p.Gln501Lys | missense variant | rs10192036 | 0.405 |
| R/D | IL1RL1 | 2 | 102351896 | C | T | ENST00000233954.5:c.1646C>T | ENSP00000233954.1:p.Thr549Ile | missense variant | rs10192157 | 0.405 |
| R/D | IL1RL1 | 2 | 102351752 | A | G | ENST00000233954.5:c.1502A>G | ENSP00000233954.1:p.Gln501Arg | missense variant | rs10204137 | 0.405 |
| R/D | IL1RL1 | 2 | 102351902 | T | C | ENST00000233954.5:c.1652T>C | ENSP00000233954.1:p.Leu551Ser | missense variant | rs10206753 | 0.405 |
| R/D | IL1RL1 | 2 | 102339008 | C | A | ENST00000233954.5:c.233C>A | ENSP00000233954.1:p.Ala78Glu | missense variant | rs1041973 | 0.237 |
| R/D | IL1RL1 | 2 | 102351547 | G | A | ENST00000233954.5:c.1297G>A | ENSP00000233954.1:p.Ala433Thr | missense variant | rs4988956 | 0.405 |
| R/D | IL1RL2 | 2 | 102235248 | T | C | ENST00000264257.6:c.1649T>C | ENSP00000264257.2:p.Leu550Pro | missense variant | rs2302612 | 0.189 |
| R/D | IL1RN | 2 | 113129906 | C | T | ENST00000259206.9:c.214+242C>T |  | intron variant | rs2071459 | 0.137 |
| R/D | IL1RN | 2 | 113129758 | T | G | ENST00000259206.9:c.214+94T>G |  | intron variant | rs2232354 | 0.204 |
| R/D | IL1RN | 2 | 113119446 | A | G | ENST00000259206.9:c.11-620G>A |  | intron variant | rs2592346 | 0.428 |
| R/D | IL1RN | 2 | 113119202 | A | G | ENST00000259206.9:c.11-864G>A |  | intron variant | rs2637988 | 0.429 |
| R/D | IL1RN | 2 | 113129881 | G | T | ENST00000259206.9:c.214+217G>T |  | intron variant | rs408392 | 0.292 |
| R/D | IL1RN | 2 | 113129685 | C | T | ENST00000259206.9:c.214+21C>T |  | intron variant | rs423904 | 0.292 |
| R/D | IL1RN | 2 | 113118176 | C | G | ENST00000259206.9:c.10+148C>G |  | intron variant | rs4251969 | 0.290 |
| R/D | IL1RN | 2 | 113118196 | A | G | ENST00000259206.9:c.10+168A>G |  | intron variant | rs4251970 | 0.290 |
| R/D | IL1RN | 2 | 113118490 | G | A | ENST00000259206.9:c.10+462A>G |  | intron variant | rs4251972 | 0.281 |
| R/D | IL1RN | 2 | 113118501 | C | G | ENST00000259206.9:c.10+473C>G |  | intron variant | rs4251974 | 0.289 |
| R/D | IL1RN | 2 | 113118583 | A | G | ENST00000259206.9:c.10+555A>G |  | intron variant | rs4251975 | 0.289 |
| R/D | IL1RN | 2 | 113118603 | C | A | ENST00000259206.9:c.10+575C>A |  | intron variant | rs4251976 | 0.289 |
| R/D | IL1RN | 2 | 113119125 | G | A | ENST00000259206.9:c.11-941G>A |  | intron variant | rs4251981 | 0.289 |
| R/D | IL1RN | 2 | 113119442 | A | G | ENST00000259206.9:c.11-624A>G |  | intron variant | rs4251983 | 0.288 |
| R/D | IL1RN | 2 | 113119649 | A | G | ENST00000259206.9:c.11-417A>G |  | intron variant | rs4251984 | 0.289 |
| R/D | IL1RN | 2 | 113119836 | G | T | ENST00000259206.9:c.11-230G>T |  | intron variant | rs4251985 | 0.288 |
| R/D | IL1RN | 2 | 113124439 | GC | G | ENST00000259206.9:c.74-3241delC |  | intron variant | rs4251999 | 0.433 |
| R/D | IL1RN | 2 | 113126824 | G | A | ENST00000259206.9:c.74-865G>A |  | intron variant | rs439154 | 0.428 |
| R/D | IL1RN | 2 | 113129822 | G | A | ENST00000259206.9:c.214+158G>A |  | intron variant | rs442710 | 0.292 |
| R/D | IL1RN | 2 | 113129696 | G | A | ENST00000259206.9:c.214+32G>A |  | intron variant | rs446433 | 0.292 |
| R/D | IL1RN | 2 | 113130095 | A | G | ENST00000259206.9:c.214+431A>G |  | intron variant | rs447713 | 0.292 |
| R/D | IL1RN | 2 | 113129717 | G | C | ENST00000259206.9:c.214+53G>C |  | intron variant | rs495282 | 0.292 |
| R/D | IL1RN | 2 | 113129761 | A | C | ENST00000259206.9:c.214+97A>C |  | intron variant | rs495410 | 0.292 |
| R/D | IL1RN | 2 | 113120136 | A | C | ENST00000259206.9:c.73+8A>C |  | splice region variant | rs878972 | 0.289 |
| R/D | IL2 | 4 | 122454980 | G | A | ENST00000226730.4:c.208-1127C>T |  | intron variant | rs2069778 | 0.151 |
| R/D | IL2RA | 10 | 6024237 | C | G | ENST00000379959.7:c.367+7G>C |  | splice region variant | rs11256369 | 0.231 |
| R/D | IL2RB | 22 | 37128579 | G | T | ENST00000216223.9:c.1173C>A | ENSP00000216223.5:p.Asp391Glu | missense variant | rs228942 | 0.184 |
| R/D | IL2RB | 22 | 37136401 | G | A | ENST00000216223.9:c.538-8C>T |  | splice region variant | rs3218297 | 0.126 |
| R/D | IL4 | 5 | 132675473 | GC | G | ENST00000231449.6:c.183+969delC |  | intron variant | rs11479198 | 0.168 |
| R/D | IL4 | 5 | 132677487 | G | C | ENST00000231449.6:c.184-2227C>G |  | intron variant | rs2227282 | 0.276 |
| R/D | IL4 | 5 | 132677033 | G | T | ENST00000231449.6:c.183+2527T>G |  | intron variant | rs2227284 | 0.276 |
| R/D | IL4 | 5 | 132677607 | G | C | ENST00000231449.6:c.184-2107C>G |  | intron variant | rs2243263 | 0.100 |
| R/D | IL4 | 5 | 132678097 | G | A | ENST00000231449.6:c.184-1617G>A |  | intron variant | rs2243266 | 0.168 |
| R/D | IL4 | 5 | 132678194 | G | C | ENST00000231449.6:c.184-1520G>C |  | intron variant | rs2243267 | 0.169 |
| R/D | IL4 | 5 | 132678271 | A | C | ENST00000231449.6:c.184-1443A>C |  | intron variant | rs2243268 | 0.167 |
| R/D | IL4 | 5 | 132678319 | TAA | T | ENST00000231449.6:c.184-1393_184-1392delAA |  | intron variant | rs2243269 | 0.168 |
| R/D | IL4 | 5 | 132678417 | A | G | ENST00000231449.6:c.184-1297A>G |  | intron variant | rs2243270 | 0.176 |
| R/D | IL4 | 5 | 132680862 | C | A | ENST00000231449.6:c.360+972C>A |  | intron variant | rs2243282 | 0.169 |
| R/D | IL4 | 5 | 132681300 | G | A | ENST00000231449.6:c.361-1186G>A |  | intron variant | rs2243284 | 0.177 |
| R/D | IL4 | 5 | 132681301 | G | T | ENST00000231449.6:c.361-1185T>G |  | intron variant | rs2243285 | 0.100 |
| R/D | IL4 | 5 | 132675034 | C | T | ENST00000231449.6:c.183+528C>T |  | intron variant | rs734244 | 0.168 |
| R/D | IL4R | 16 | 27363079 | A | G | ENST00000395762.6:c.1727A>G | ENSP00000379111.2:p.Gln576Arg | missense variant | rs1801275 | 0.208 |
| R/D | IL4R | 16 | 27344882 | A | G | ENST00000395762.6:c.223A>G | ENSP00000379111.2:p.Ile75Val | missense variant | rs1805010 | 0.425 |
| R/D | IL4R | 16 | 27362551 | A | C | ENST00000395762.6:c.1199A>C | ENSP00000379111.2:p.Glu400Ala | missense variant | rs1805011 | 0.106 |
| R/D | IL4R | 16 | 27362643 | T | C | ENST00000395762.6:c.1291T>C | ENSP00000379111.2:p.Cys431Arg | missense variant | rs1805012 | 0.102 |
| R/D | IL4R | 16 | 27362859 | T | C | ENST00000395762.6:c.1507T>C | ENSP00000379111.2:p.Ser503Pro | missense variant | rs1805015 | 0.152 |
| R/D | IL6 | 7 | 22728505 | C | A | ENST00000404625.5:c.211-188C>A |  | intron variant | rs1474347 | 0.485 |
| R/D | IL6 | 7 | 22728289 | C | G | ENST00000404625.5:c.211-404C>G |  | intron variant | rs1474348 | 0.485 |
| R/D | IL6 | 7 | 22729088 | A | G | ENST00000404625.5:c.324+282T>G |  | intron variant | rs1554606 | 0.470 |
| R/D | IL6 | 7 | 22727026 | C | G | ENST00000404625.5:c.-84-153C>G |  | intron variant | rs1800795 | 0.485 |
| R/D | IL6 | 7 | 22726602 | A | G | ENST00000404625.5:c.-85+344A>G |  | intron variant | rs1800797 | 0.470 |
| R/D | IL6 | 7 | 22727814 | A | G | ENST00000404625.5:c.210+180A>G |  | intron variant | rs2069832 | 0.485 |
| R/D | IL6 | 7 | 22728953 | C | G | ENST00000404625.5:c.324+147C>G |  | intron variant | rs2069840 | 0.332 |
| R/D | IL6 | 7 | 22730530 | G | T | ENST00000404625.5:c.471+870G>A |  | intron variant | rs2069845 | 0.433 |
| R/D | IL6 | 7 | 22726814 | T | A | ENST00000404625.5:c.-84-365T>A |  | intron variant | rs7802307 | 0.495 |
| R/D | IL6 | 7 | 22726815 | T | A | ENST00000404625.5:c.-84-364T>A |  | intron variant | rs7802308 | 0.191 |
| R/D | IL6R | 1 | 154454494 | A | C | ENST00000368485.7:c.1073A>C | ENSP00000357470.3:p.Asp358Ala | missense variant | rs2228145 | 0.360 |
| R/D | IL7R | 5 | 35875491 | T | A | ENST00000303115.7:c.801-21A>T |  | intron variant | rs987106 | 0.445 |
| R/D | IL7R | 5 | 35871088 | A | G | ENST00000303115.7:c.412G>A | ENSP00000306157.3:p.Val138Ile | missense variant | rs1494555 | 0.296 |
| R/D | IL7R | 5 | 35860966 | C | T | ENST00000303115.7:c.197T>C | ENSP00000306157.3:p.Ile66Thr | missense variant | rs1494558 | 0.302 |
| R/D | IL7R | 5 | 35876172 | A | G | ENST00000303115.7:c.1066A>G | ENSP00000306157.3:p.Ile356Val | missense variant | rs3194051 | 0.282 |
| R/D | IL7R | 5 | 35874473 | C | T | ENST00000303115.7:c.731C>T | ENSP00000306157.3:p.Thr244Ile | missense variant | rs6897932 | 0.271 |
| R/D | KIR2DL1 | 19 | 54771302 | G | A | ENST00000336077.10:c.70+418G>A |  | intron variant | rs376251430 | 0.157 |
| R/D | KIR2DL1 | 19 | 54775198 | T | C | ENST00000336077.10:c.404T>C | ENSP00000336769.5:p.Leu135Pro | missense variant | rs11673144 | 0.443 |
| R/D | KIR2DL1 | 19 | 54769863 | G | T | ENST00000336077.10:c.13G>T | ENSP00000336769.5:p.Val5Phe | missense variant | rs2304224 | 0.306 |
| R/D | KIR2DL1 | 19 | 54783002 | C | T | ENST00000336077.10:c.796C>T | ENSP00000336769.5:p.Arg266Cys | missense variant | rs34721508 | 0.154 |
| R/D | KIR2DL3 | 19 | 54747356 | C | T | ENST00000342376.3:c.686C>T | ENSP00000342215.3:p.Pro229Leu | missense variant | rs35861855 | 0.284 |
| R/D | KIR2DS4 | 19 | 54837818 | C | A | ENST00000339924.12:c.313C>A | ENSP00000340011.8:p.Pro105Thr | missense variant | rs1130492 | 0.189 |
| R/D | KIR2DS4 | 19 | 54839524 | A | G | ENST00000339924.12:c.467A>G | ENSP00000340011.8:p.Lys156Arg | missense variant | rs4806590 | 0.290 |
| R/D | KIR2DS4 | 19 | 54837576 | G | A | ENST00000339924.12:c.71G>A | ENSP00000340011.8:p.Gly24Glu | missense variant | rs58731871 | 0.189 |
| R/D | KIR3DL1 | 19 | 54818467 | A | T | ENST00000391728.8:c.223A>T | ENSP00000375608.4:p.Ile75Leu | missense variant | rs1049150 | 0.371 |
| R/D | KIR3DL1 | 19 | 54821684 | G | C | ENST00000391728.8:c.775G>C | ENSP00000375608.4:p.Gly259Arg | missense variant | rs1049215 | 0.146 |
| R/D | KIR3DL1 | 19 | 54830120 | G | C | ENST00000391728.8:c.1180G>C | ENSP00000375608.4:p.Glu394Gln | missense variant | rs1130513 | 0.294 |
| R/D | KIR3DL1 | 19 | 54818564 | C | T | ENST00000391728.8:c.320C>T | ENSP00000375608.4:p.Ser107Leu | missense variant | rs143159382 | 0.242 |
| R/D | KIR3DL1 | 19 | 54818399 | G | A | ENST00000391728.8:c.155G>A | ENSP00000375608.4:p.Arg52His | missense variant | rs144994606 | 0.225 |
| R/D | KIR3DL1 | 19 | 54818437 | A | G | ENST00000391728.8:c.193A>G | ENSP00000375608.4:p.Arg65Gly | missense variant | rs149123986 | 0.244 |
| R/D | KIR3DL1 | 19 | 54819964 | C | T | ENST00000391728.8:c.607C>T | ENSP00000375608.4:p.Pro203Ser | missense variant | rs2273731 | 0.428 |
| R/D | KIR3DL1 | 19 | 54821820 | G | T | ENST00000391728.8:c.911G>T | ENSP00000375608.4:p.Trp304Leu | missense variant | rs35974949 | 0.429 |
| R/D | KIR3DL1 | 19 | 54829451 | G | A | ENST00000391728.8:c.1091G>A | ENSP00000375608.4:p.Cys364Tyr | missense variant | rs45542639 | 0.248 |
| R/D | KIR3DL1 | 19 | 54829381 | A | G | ENST00000391728.8:c.1021A>G | ENSP00000375608.4:p.Ile341Val | missense variant | rs45551936 | 0.299 |
| R/D | KIR3DL1 | 19 | 54816505 | C | T | ENST00000391728.8:c.5C>T | ENSP00000375608.4:p.Ser2Leu | missense variant | rs605219 | 0.174 |
| R/D | KIR3DL1 | 19 | 54818446 | A | G | ENST00000391728.8:c.202A>G | ENSP00000375608.4:p.Ile68Val | missense variant | rs643347 | 0.369 |
| R/D | KIR3DL2 | 19 | 54853865 | G | T | ENST00000326321.7:c.474G>T | ENSP00000325525.3:p.Glu158Asp | missense variant | rs1048270 | 0.217 |
| R/D | KIR3DL2 | 19 | 54852264 | C | G | ENST00000326321.7:c.337C>G | ENSP00000325525.3:p.Leu113Val | missense variant | rs3188286 | 0.268 |
| R/D | KIR3DL2 | 19 | 54866553 | C | T | ENST00000326321.7:c.1190C>T | ENSP00000325525.3:p.Thr397Met | missense variant | rs3745902 | 0.300 |
| R/D | KIR3DL3 | 19 | 54727239 | A | G | ENST00000291860.1:c.356-372G>A |  | intron variant | rs34614411 | 0.265 |
| R/D | KIR3DL3 | 19 | 54727164 | C | A | ENST00000291860.1:c.356-447A>C |  | intron variant | rs34830246 | 0.282 |
| R/D | KIR3DL3 | 19 | 54727369 | C | A | ENST00000291860.1:c.356-242C>A |  | intron variant | rs34876880 | 0.449 |
| R/D | KIR3DL3 | 19 | 54727502 | G | C | ENST00000291860.1:c.356-109C>G |  | intron variant | rs35093205 | 0.405 |
| R/D | KIR3DL3 | 19 | 54727323 | C | T | ENST00000291860.1:c.356-288C>T |  | intron variant | rs35508109 | 0.172 |
| R/D | KIR3DL3 | 19 | 54727304 | G | A | ENST00000291860.1:c.356-307A>G |  | intron variant | rs35910454 | 0.302 |
| R/D | KIR3DL3 | 19 | 54726137 | G | A | ENST00000291860.1:c.155G>A | ENSP00000291860.1:p.Arg52His | missense variant | rs113988102 | 0.305 |
| R/D | KIR3DL3 | 19 | 54735264 | C | A | ENST00000291860.1:c.961A>C | ENSP00000291860.1:p.Asn321His | missense variant | rs602444 | 0.480 |
| R/D | KIR3DL3 | 19 | 54727702 | G | T | ENST00000291860.1:c.447G>T | ENSP00000291860.1:p.Arg149Ser | missense variant | rs62132665 | 0.105 |
| D | LTA | 6 | 31573007 | C | A | ENST00000454783.5:c.179C>A | ENSP00000403495.1:p.Thr60Asn | missense variant | rs1041981 | 0.306 |
| D | LTA | 6 | 31572779 | T | C | ENST00000454783.5:c.37T>C | ENSP00000403495.1:p.Cys13Arg | missense variant | rs2229094 | 0.287 |
| R | TGFB1 | 19 | 41346210 | C | T | ENST00000221930.5:c.517-1346A>G |  | intron variant | rs1989457 | 0.426 |
| R/D | TGFB1 | 19 | 41342943 | G | A | ENST00000221930.5:c.635-696T>C |  | intron variant | rs10416269 | 0.417 |
| R/D | TGFB1 | 19 | 41339408 | C | T | ENST00000221930.5:c.860+2475G>A |  | intron variant | rs11466344 | 0.218 |
| R/D | TGFB1 | 19 | 41337556 | T | C | ENST00000221930.5:c.860+4327A>G |  | intron variant | rs11466345 | 0.103 |
| R/D | TGFB1 | 19 | 41351499 | C | T | ENST00000221930.5:c.355+1191A>G |  | intron variant | rs12462166 | 0.315 |
| R/D | TGFB1 | 19 | 41351534 | A | G | ENST00000221930.5:c.355+1156C>T |  | intron variant | rs12983775 | 0.315 |
| R/D | TGFB1 | 19 | 41351804 | TGGGGGGGCTCGCTGCTC | T | ENST00000221930.5:c.355+885_355+886insGAGCAGCGAGCCCCCCC |  | intron variant | rs150460951 | 0.316 |
| R/D | TGFB1 | 19 | 41350981 | C | A | ENST00000221930.5:c.355+1709T>G |  | intron variant | rs2241715 | 0.314 |
| R/D | TGFB1 | 19 | 41348147 | A | C | ENST00000221930.5:c.516+148G>T |  | intron variant | rs2241717 | 0.431 |
| R/D | TGFB1 | 19 | 41339853 | C | G | ENST00000221930.5:c.860+2030G>C |  | intron variant | rs2278422 | 0.427 |
| R/D | TGFB1 | 19 | 41333726 | T | C | ENST00000221930.5:c.861-1445A>G |  | intron variant | rs8105161 | 0.168 |
| R/D | TGFB1 | 19 | 41348629 | A | T | ENST00000221930.5:c.356-174A>T |  | intron variant | rs8108632 | 0.433 |
| R/D | TGFB1 | 19 | 41353016 | A | G | ENST00000221930.5:c.29C>T | ENSP00000221930.4:p.Pro10Leu | missense variant | rs1800470 | 0.382 |
| R/D | TGFB2 | 1 | 218347501 | G | A | ENST00000366929.4:c.346+454G>A |  | intron variant | rs10482724 | 0.163 |
| R/D | TGFB2 | 1 | 218402545 | A | G | ENST00000366929.4:c.431-2624A>G |  | intron variant | rs10746379 | 0.134 |
| R/D | TGFB2 | 1 | 218350962 | A | G | ENST00000366929.4:c.346+3915A>G |  | intron variant | rs11581605 | 0.226 |
| R/D | TGFB2 | 1 | 218401860 | G | A | ENST00000366929.4:c.431-3309G>A |  | intron variant | rs1317681 | 0.166 |
| R/D | TGFB2 | 1 | 218350388 | C | T | ENST00000366929.4:c.346+3341C>T |  | intron variant | rs1417488 | 0.249 |
| R/D | TGFB2 | 1 | 218350308 | G | A | ENST00000366929.4:c.346+3261G>A |  | intron variant | rs1418555 | 0.225 |
| R/D | TGFB2 | 1 | 218409436 | T | C | ENST00000366929.4:c.594+4104T>C |  | intron variant | rs17047804 | 0.127 |
| R/D | TGFB2 | 1 | 218375179 | C | T | ENST00000366929.4:c.430+11762C>T |  | intron variant | rs17558745 | 0.295 |
| R/D | TGFB2 | 1 | 218380187 | C | T | ENST00000366929.4:c.430+16770C>T |  | intron variant | rs2009112 | 0.384 |
| R/D | TGFB2 | 1 | 218384985 | A | C | ENST00000366929.4:c.431-20184C>A |  | intron variant | rs2027566 | 0.373 |
| R/D | TGFB2 | 1 | 218415855 | T | C | ENST00000366929.4:c.594+10523T>C |  | intron variant | rs2796813 | 0.425 |
| R/D | TGFB2 | 1 | 218377666 | T | G | ENST00000366929.4:c.430+14249T>G |  | intron variant | rs2796817 | 0.150 |
| R/D | TGFB2 | 1 | 218407028 | A | G | ENST00000366929.4:c.594+1696A>G |  | intron variant | rs2796819 | 0.435 |
| R/D | TGFB2 | 1 | 218348267 | A | G | ENST00000366929.4:c.346+1220G>A |  | intron variant | rs2799098 | 0.197 |
| R/D | TGFB2 | 1 | 218379955 | A | G | ENST00000366929.4:c.430+16538A>G |  | intron variant | rs3892225 | 0.185 |
| R/D | TGFB2 | 1 | 218416124 | TG | T | ENST00000366929.4:c.594+10797delG |  | intron variant | rs5781034 | 0.197 |
| R/D | TGFB2 | 1 | 218398415 | A | T | ENST00000366929.4:c.431-6754A>T |  | intron variant | rs61823428 | 0.128 |
| R/D | TGFB2 | 1 | 218347653 | A | G | ENST00000366929.4:c.346+606A>G |  | intron variant | rs6658835 | 0.269 |
| R/D | TGFB2 | 1 | 218384123 | G | GA | ENST00000366929.4:c.430+20717delA |  | intron variant | rs74882313 | 0.417 |
| R/D | TNF | 6 | 31576412 | A | G | ENST00000449264.2:c.187-122A>G |  | intron variant | rs3093662 | 0.108 |

R: recipient; D: donor; Chr: chromosome; HGVSc: Human Genome Variation Society (HGVS) coding sequence name; HGVSp: HGVS protein sequence name; dbSNP: Single Nucleotide Polymorphism Database**;** MAF**:** minor allele frequency.

**Supplementary table 3.** Summary of the polymorphisms selected in each of the models constructed to predict CMV reactivation after allo-HSCT.

| **Model** | **SNPs** | **AUC** | **Se (%)** | **Sp (%)** |
| --- | --- | --- | --- | --- |
| m1 | rs2243123 (R) | 0.637 | 80.4 | 47.1 |
| m2 | rs2243123 (R) rs3093662 (D) | 0.708 | 80.4 | 47.1 |
| m3 | rs2839695 (R) rs144994606 (R) rs5781034 (R) | 0.760 | 86.3 | 41.2 |
| m4 | rs2839695 (R) rs144994606 (R) rs5781034 (R) rs2295615 (D) | 0.790 | 86.3 | 58.8 |
| m5 | rs2839695 (R) rs7615589(R) rs45542639(R) rs5781034(R) rs2295615(D) | 0.814 | 82.4 | 67.6 |
| m6 | rs2839695(R) rs2243123(R) rs143159382(R) rs5781034(R) rs3093662(D) rs2295615(D) | 0.824 | 84.3 | 58.8 |
| m7 | rs2839695(R) rs2243123(R) rs149123986(R) rs5781034(R) rs3093662(D) rs439154(D) rs2295615(D) | 0.826 | 80.4 | 64.7 |
| m8 | rs2839695(R) rs2243123(R) rs45542639(R) rs144994606(R) rs5781034(R) rs3093662(D) rs439154(D) rs2295615(D) | 0.830 | 82.4 | 61.8 |
| m9 | rs2839695(R) rs2243123(R) rs45542639(R) rs149123986(R) rs144994606(R) rs5781034(R) rs3093662(D) rs439154(D) rs2295615(D) | 0.830 | 82.4 | 61.8 |
| m10 | rs2839695(R) rs2243123(R) rs149123986(R) rs143159382(R) rs144994606(R) rs5781034(R) rs3093662(R) rs3093662(D) rs439154(D) rs2295615(D) | 0.832 | 80.4 | 67.6 |
| m11 | rs2839695(R) rs7615589(R) rs2243123(R) rs45542639(R) rs143159382(R) rs144994606(R) rs5781034(R) rs3093662(R) rs3093662(D) rs439154(D) rs2295615(D) | 0.833 | 82.4 | 67.6 |
| m12 | rs2839695(R) rs7615589(R) rs2243123(R) rs45542639(R) rs149123986(R) rs143159382(R) rs144994606(R) rs5781034(R) rs3093662(R) rs3093662(D) rs439154(D) rs2295615(D) | 0.833 | 82.4 | 67.6 |

SNP: Single Nucleotide Polymorphism; R: recipient; D: donor; AUC: area under the curve; Se: sensibility; Sp: specificity.

**Supplementary table 4.** Statistical parameters calculated for each of the cut-off values to evaluate the clinical utility of the genetic risk scores constructed. A model with five genetic variants (CXCL12 rs2839695, IL12A rs7615589, KIR3DL1 rs4554639, TGFB2 rs5781034 for the recipient and CD48 rs2295615 for the donor) was selected (highlighted in yellow) for the analysis.

|  |  |  |  |  | **Likelihood Ratios (LR)** | | **Predictive Values (PV)** | |
| --- | --- | --- | --- | --- | --- | --- | --- | --- |
| **Cut-off** | **Se (%)** | **Sp (%)** | **Eff(%)** | **Youden Index (%)** | **LR+** | **1/LR-** | **PV+(%)** | **PV-(%)** |
| ≥ 0.061059 | 100.0 | 0.0 | 60.0 | 0.0 | 1.00 | . | 60.00 | . |
| ≥ 0.139523 | 98.0 | 2.9 | 60.0 | 1 | 1.01 | 1.5 | 60.24 | 50.00 |
| ≥ 0.168986 | 98.0 | 8.8 | 62.4 | 6.9 | 1.08 | 4.50 | 61.73 | 75.00 |
| ≥ 0.219672 | 98.0 | 17.6 | 65.9 | 15.7 | 1.19 | 9.00 | 64.10 | 85.71 |
| ≥ 0.334667 | 96.1 | 32.4 | 70.6 | 28.4 | 1.42 | 8.25 | 68.06 | 84.62 |
| ≥ 0.336445 | 96.1 | 35.3 | 71.8 | 31.4 | 1.48 | 9.00 | 69.01 | 85.71 |
| ≥ 0.386814 | 90.2 | 41.2 | 70.6 | 31.4 | 1.53 | 4.20 | 69.70 | 73.68 |
| ≥ 0.409695 | 90.2 | 47.1 | 72.9 | 37.3 | 1.70 | 4.80 | 71.88 | 76.19 |
| ≥ 0.412432 | 90.2 | 52.9 | 75.3 | 43.1 | 1.92 | 5.40 | 74.19 | 78.26 |
| ≥ 0.468169 | 88.2 | 58.8 | 76.5 | 47.1 | 2.14 | 5.00 | 76.27 | 76.92 |
| ≥ 0.490008 | 84.3 | 67.6 | 77.6 | 52.0 | 2.61 | 4.31 | 79.63 | 74.19 |
| ≥ 0.611336 | 82.4 | 67.6 | 76.5 | 50.0 | 2.55 | 3.83 | 79.25 | 71.88 |
| ≥ 0.631919 | 74.5 | 70.6 | 72.9 | 45.1 | 2.53 | 2.77 | 79.17 | 64.86 |
| ≥ 0.633772 | 74.5 | 73.5 | 74.1 | 48.0 | 2.81 | 2.88 | 80.85 | 65.79 |
| ≥ 0.682846 | 72.5 | 76.5 | 74.1 | 49.0 | 3.08 | 2.79 | 82.22 | 65.00 |
| ≥ 0.687007 | 70.6 | 76.5 | 72.9 | 47.1 | 3.00 | 2.60 | 81.82 | 63.41 |
| ≥ 0.705512 | 51.0 | 91.2 | 67.1 | 42.2 | 5.78 | 1.86 | 89.66 | 55.36 |
| ≥ 0.731965 | 49.0 | 91.2 | 65.9 | 40.2 | 5.56 | 1.79 | 89.29 | 54.39 |
| ≥ 0.750281 | 43.1 | 91.2 | 62.4 | 34.3 | 4.89 | 1.60 | 88.00 | 51.67 |
| ≥ 0 .842976 | 41.2 | 91.2 | 61.2 | 32.4 | 4.67 | 1.55 | 87.50 | 50.82 |
| ≥ 0.871945 | 39.2 | 91.2 | 60.0 | 30.4 | 4.44 | 1.50 | 86.96 | 50.00 |
| ≥ 0.881404 | 25.5 | 97.1 | 54.1 | 22.5 | 8.67 | 1.30 | 92.86 | 46.48 |
| ≥ 0.882235 | 23.5 | 97.1 | 52.9 | 20.6 | 8.00 | 1.27 | 92.31 | 45.83 |
| ≥ 0.958746 | 11.8 | 100.0 | 47.1 | 11.8 | . | 1.13 | 100.00 | 43.04 |
| > 0.958746 | 0.0 | 100.0 | 40.0 | 0.0 | . | 1.00 | . | 40.00 |

Se: sensibility; Sp: specificity; Eff: effectiveness.

**Supplementary table 5. Risk score of the whole cohort of patients using the proposed predictive model.** Four patients (highlighted in yellow) stratified as low-risk of CMV infection (<0.49) with the predict model at pre-transplantation suffered grade III-IV aGVHD and were re-stratified to high risk.

| **Patients** | **Pre-transplant score** | **Grade III/IV aGVHD^a^** |
| --- | --- | --- |
| 1 | 0.87 | 1 |
| 2 | 0.47 | 0 |
| 3 | 0.87 | 0 |
| 4 | 0.47 | 0 |
| 5 | 0.69 | 0 |
| 6 | 0.87 | 0 |
| 7 | 0.14 | 0 |
| 8 | 0.47 | 0 |
| 9 | 0.69 | 0 |
| 10 | 0.87 | 1 |
| 11 | 0.14 | 0 |
| 12 | 0.47 | 1 |
| 13 | 0.61 | 0 |
| 14 | 0.69 | 0 |
| 15 | 0.87 | 1 |
| 16 | 0.47 | 1 |
| 17 | 0.61 | 1 |
| 18 | 0.69 | 0 |
| 19 | 0.87 | 0 |
| 20 | 0.88 | 1 |
| 21 | 0.34 | 0 |
| 22 | 0.49 | 1 |
| 23 | 0.61 | 0 |
| 24 | 0.69 | 0 |
| 25 | 0.87 | 1 |
| 26 | 0.88 | 0 |
| 27 | 0.34 | 0 |
| 28 | 0.61 | 0 |
| 29 | 0.69 | 0 |
| 30 | 0.73 | 1 |
| 31 | 0.87 | 0 |
| 32 | 0.88 | 0 |
| 33 | 0.17 | 0 |
| 34 | 0.34 | 1 |
| 35 | 0.61 | 0 |
| 36 | 0.69 | 0 |
| 37 | 0.73 | 0 |
| 38 | 0.87 | 0 |
| 39 | 0.88 | 1 |
| 40 | 0.17 | 0 |
| 41 | 0.34 | 0 |
| 42 | 0.63 | 0 |
| 43 | 0.69 | 0 |
| 44 | 0.73 | 1 |
| 45 | 0.88 | 0 |
| 46 | 0.88 | 0 |
| 47 | 0.17 | 0 |
| 48 | 0.34 | 0 |
| 49 | 0.63 | 0 |
| 50 | 0.69 | 0 |
| 51 | 0.75 | 1 |
| 52 | 0.88 | 0 |
| 53 | 0.22 | 0 |
| 54 | 0.39 | 0 |
| 55 | 0.63 | 0 |
| 56 | 0.69 | 0 |
| 57 | 0.84 | 0 |
| 58 | 0.88 | 0 |
| 59 | 0.22 | 0 |
| 60 | 0.39 | 0 |
| 61 | 0.68 | 1 |
| 62 | 0.69 | 0 |
| 63 | 0.96 | 0 |
| 64 | 0.22 | 0 |
| 65 | 0.41 | 0 |
| 66 | 0.69 | 0 |
| 67 | 0.96 | 0 |
| 68 | 0.22 | 0 |
| 69 | 0.41 | 0 |
| 70 | 0.69 | 0 |
| 71 | 0.96 | 0 |
| 72 | 0.22 | 0 |
| 73 | 0.41 | 0 |
| 74 | 0.69 | 1 |
| 75 | 0.96 | 1 |
| 76 | 0.22 | 1 |
| 77 | 0.41 | 0 |
| 78 | 0.69 | 0 |
| 79 | 0.96 | 1 |
| 80 | 0.33 | 0 |
| 81 | 0.41 | 0 |
| 82 | 0.71 | 1 |
| 83 | 0.96 | 0 |
| 84 | 0.06 | 0 |
| 85 | 0.06 | 0 |

^a^0: no aGVHD grade III/IV; 1: patients with grade III/IV aGVHD post-transplantation
